# Retinal imaging with optical coherence tomography in multiple sclerosis: novel aspects

**DOI:** 10.1007/s10354-022-00925-2

**Published:** 2022-03-28

**Authors:** Elisabeth Olbert, Walter Struhal

**Affiliations:** 1 Department of Neurology, University Hospital Tulln, Alter Ziegelweg 10, 3430 Tulln an der Donau, Austria; 2 Karl Landsteiner University of Health Sciences, Tulln, Austria

**Keywords:** Multiple sclerosis, Optic neuritis, Retina, Macula, Atrophy

## Abstract

Optical coherence tomography (OCT) is of increasing interest in the clinical assessment of multiple sclerosis (MS) patients beyond the scope of clinical studies. In this narrative review, we discuss novel changes of OCT parameters during acute optic neuritis and the disease course of MS patients. OCT images document the changes of retinal layers during an episode of acute optic neuritis and can therefore provide valuable insights into the pathophysiology. Moreover, MS patients show progredient thinning of retinal layers throughout the disease. The thinning is accelerated through relapses as well as disease progression without relapse. The OCT parameters are also associated with clinical outcome parameters, including disability, cognitive function, and brain atrophy. The impact of disease-modifying therapies on OCT parameters is the subject of ongoing research and depends on the agent used. Additional data are still necessary before OCT parameters can be implemented in the clinical standard of care of MS patients.

## Abstract (de)

Die optische Kohärenztomographie (OCT) ist, auch außerhalb von klinischen Studien, von zunehmender Bedeutung für die klinische Behandlung von Patienten mit multipler Sklerose (MS). In der vorliegenden Übersichtsarbeit werden die Veränderungen verschiedener OCT-Parameter während einer akuten Entzündung des Sehnervs sowie im Verlauf einer MS-Erkrankung erörtert. Während einer akuten Entzündung des Sehnervs zeigt die OCT die Veränderungen der Schichten der Retina. Hieraus können neue pathophysiologische Erkenntnisse zur MS-Erkrankung gewonnen werden. Im Krankheitsverlauf zeigen MS-Patienten eine Abnahme der Dicke von retinalen Schichten, die durch Schubaktivität und progrediente Verschlechterung verstärkt wird. Die OCT-Parameter stehen in Korrelation zu der Schwere der körperlichen Behinderung, kognitiver Leistungsfähigkeit sowie Gehirnatrophie. MS-Basistherapien vermindern die Atrophie der retinalen Schichten und haben einen positiven Einfluss auf die Schichtdicke. Daher werden OCT-Parameter zunehmend als zusätzliche Outcome-Parameter in klinischen Studien verwendet. Weitere Studien sind notwendig, bevor OCT-Untersuchungen in die Standardversorgung von MS-Patienten aufgenommen werden können.

## Introduction

Optical coherence tomography (OCT) is a noninvasive method that ophthalmologists routinely use to examine the retina. It is part of the clinical routine in the medical care of patients with macula diseases. OCT is also used to study the retina in patients with optic neuritis (ON), multiple sclerosis (MS), and neurodegenerative diseases. OCT could provide additional information for neuroimmunologists in the care of MS patients and help solve common clinical challenges in the future. These include the need to objectify a reported previous ON episode, differentiate between different demyelinating diseases, and give additional clues to predict disease course in MS patients. The purpose of this paper is to review novel aspects of OCT parameters in MS patients with an emphasis on new literature published in 2020 and 2021. This review focuses on: (i) OCT changes in acute ON, (ii) the detection of prior ON episodes, (iii) OCT and (iv) OCT angiography changes in MS patients, and (v) the effect of disease-modifying therapy on OCT parameters.

## Optical coherence tomography

**Fig. 1:**
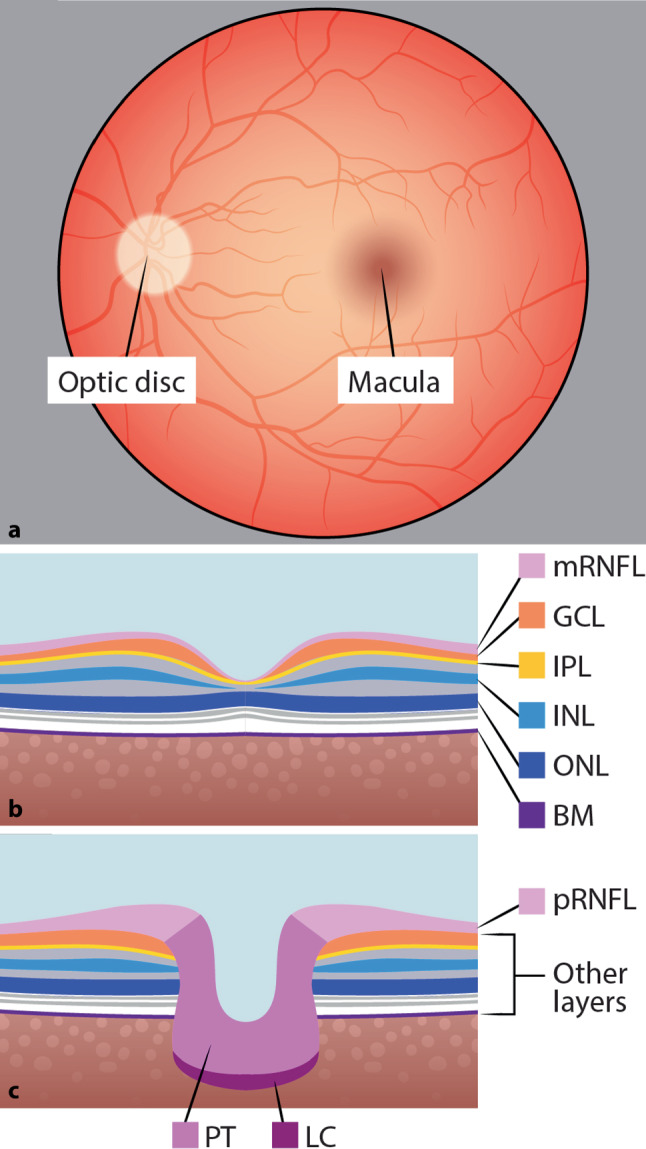
**a** Background of the eye with the macula and optic disc, **b** optical coherence tomography (OCT) of the macula, **c** OCT of the peripapillary region. *mRNFL* macula retinal fiber layer, *GCL* ganglion cell layer, *IPL* inner plexiform layer, *INL* inner nuclear layer, *ONL* outer nuclear layer, *BM* Bruch membrane, *pRNFL* peripapillary retinal fiber layer, *PT* prelaminar tissue, *LC* lamina cribrosa

OCT is a noninvasive method that yields a cross-section image of the retina by exposing the eye to near-infrared light. Specifically, an emitted light beam is scattered and reflected back by the retinal surface as well as its different layers. Each layer has distinct optic features regarding reflection, backscattering, and absorption. Knowledge about these features is then used to infer an image of the different layers of the retina and their thicknesses. An exemplary picture of an OCT scan is shown in Fig. 1.

The first generation of OCT machines were so-called time-domain OCT (TD-OCT) instruments, which used a moving reference mirror to calculate the depth (axial values) of the layers as a function of time [1]. Newer, so-called spectral-domain OCT instruments (SD-OCT) use a stationary mirror and calculate the depth using the interference spectrum. SD-OCT is more precise, less time-consuming, and exhibits a higher axial and temporal resolution. In addition, it collects more data points with each scan and thus achieves an improved signal-to-noise ratio and increased tissue contrast [2]. The newest generation is the so-called swept-source OCT (SS-OCT), promising faster scans and higher penetration. Yet, it is unclear if it attains a higher sensitivity than SD-OCT [3, 4]. To obtain a cross-section image of the retina, the light beam is moved horizontally across the retina. For volumetric scans, the cross-sections are repeated in parallel or ring-shaped patterns depending on the region of interest and the program used.

A relatively new addition to OCT imaging is OCT angiography, which can measure flow in the retinal and choroidal blood vessels without contrast-enhancing agents. Repeated OCT scans of the same area are compared by calculating the differences between the images. As a result, moving objects, such as erythrocytes, representing blood flow, become visible in a motion contrast image [4, 5].

OCT images are analyzed using manual or automated segmentation of retinal layers. Manual segmentation leads to potential bias, which needs to be considered when interpreting the results of a study [6]. Current automated segmentation techniques perform pixel-wise labeling and post-processing of the OCT image, while recent research focuses on deep learning for autosegmentation [7, 8].

In order to improve comparability between studies that use different devices and segmentation techniques, quality and reporting criteria have been developed. Quality control criteria such as OSCAR-IB, the reporting guidelines APOSTEL 2.0, and an extension of the OSCAR-IB criteria for artificial intelligence in OCT are currently used [6, 9, 10]. The APOSTEL 2.0 recommendations advise the reporting of, for example, the study protocol, OCT device, scanning protocol, quality control, and post-acquisition analysis to improve the reproducibility of OCT research. Furthermore, a consistent terminology of retinal layers, as depicted in Fig. 1, has been recommended [6].

These retinal layers include, among others, the Bruch membrane (BM), the lamina cribrosa, retinal fiber layer (RNFL) of the peripapillary (pRNFL) and macular (mRNFL) region, the inner nuclear layer (INL), the outer nuclear layer (ONL), and the ganglion cell layer (GCL) and inner plexiform layer (IPL). GCL and IPL are summarized as the ganglion cell and inner plexiform layer (GCIP), according to the new APOSTEL 2.0 criteria [6]. The vascular system of the retina consists of the superficial vascular plexus, the intermediate and deep capillary plexus, and the radial peripapillary capillary plexus and can be visualized using OCT angiography [11].

### OCT in acute optic neuritis

Changes of retinal layers during and after an acute ON episode can be used to investigate the pathophysiological mechanism of ON and provide additional information regarding location, severity, and prognosis of visual acuity. Therefore, OCT could serve as an additional tool, together with visual evoked potentials (VEP) and MRI, to objectify symptoms and the severity of an acute ON.

During an episode of acute ON, pRNFL thickness increases compared to the unaffected side, equivalent to acute edema of the retina. This effect is entirely reversed over time. PRNFL thinning is already visible 2 months after the acute ON episode and increases further with time compared to the healthy eye. This effect is accompanied by a reduction of macular thickness, especially of mRNFL and GCIP. Acute changes in GCIP within 1 month of ON onset were found to predict visual acuity 6 months after onset [12]. In addition, the inner nuclear layer (INL) thickens in the first 3 months after MS relapse, including ON. Steroid treatment can reduce this thickening. Within 1 year after the episode, INL levels return to baseline level. INL thickness is therefore discussed as a marker of acute inflammatory activity [13, 14]. INL thickening is further associated with the extent of ganglion cell loss after acute ON. A common underlying pathophysiology between INL and ganglion cell changes is discussed. One assumed mechanism for INL thickening is an accumulation of retinal fluid due to increased vascular permeability within the retina due to an injury of Müller cells. During an ON episode, GCIPL loss occurs, leading to Müller cell activation and INL thickening [14]. In addition, an effect of ON on outer retinal (OR) layers—including the outer plexiform layer, the outer nuclear layer, and the photoreceptor layer—was shown. In MS patients with ON and poor visual recovery, a thinning of OR layers, including the photoreceptor layer, was described by Ziccardi et al. However, this effect was limited to ON with poor visual outcome and was not shown in MS patients without ON or with ON but a good visual outcome. The underlying mechanism behind this effect remains unclear [15].

### Detection of prior ON episodes

Patients with an initial demyelinating event and previous symptoms consistent with ON in their medical history are a frequent challenge in clinical practice. Proving past ON episodes can change the diagnosis and, therefore, treatment options, especially in patients with clinically isolated syndrome (CIS). For this purpose, VEP has been used in the past, even though its value is limited [16]. Recent studies have investigated the merit of OCT as an alternative, yet up to this point, OCT has not been included in the diagnosis criteria of MS [17].

Different cut-off values have been proposed to discriminate between healthy eyes and eyes with previous ON. For example, inter-eye differences of ≥ 4 μm for GCIPL (sensitivity 67%, specificity 78%) and ≥ 5 μm for RNFL (sensitivity and specificity 70%, respectively) have been established by some studies [18, 19]. Other studies used cut-off values for the inter-eye GCIPL difference of 3.5 μm (sensitivity 100%, specificity 98%) and an RNFL difference of 5.5 μm (sensitivity 70%, specificity 90%) [20, 21].

Another approach included symptomatic and asymptomatic ON, defined by a lesion of the optic nerve in three-dimensional (3D) double inversion recovery (DIR) MRI. An inter-eye threshold for GCILP of ≥ 2.83 µm (symptomatic) and ≥ 1.42 µm (asymptomatic) was recommended in patients with CIS [21]. A similar study in MS patients proposed an inter-eye difference threshold of ≥ 6 µm for pRNFL (sensitivity 56.6%, specificity 86.7%) and ≥ 2.83 µm for mGCIPL (sensitivity 67.3%, specificity 67.4%). The application of DIR MRI in this cohort in the group of symptomatic ON showed a sensitivity of 84.9% and specificity of 44.4% for MRI detection of ON [22].

However, the biggest challenge in developing cut-off values is the missing gold standard to diagnose symptomatic and asymptomatic ON due to the only moderate specificity and sensitivity of currently available methods, including MRI, which is the reason for the above-described variations between studies. A possible solution to achieve acceptable sensitivity and specificity across different patient cohorts could be a combination of different OCT parameters or a combination of OCT and MRI.

Other, more unusual parameters, such as the thickness of lamina cribrosa and the Bruch’s membrane opening-minimum rim width, are of no utility for discriminating between patients with and without a history of ON [23, 24].

### Long-term OCT changes in MS patients

In addition to post ON changes, MS patients exhibit progressive neurodegenerative changes of the retina. MS and CIS patients display an annual reduction of GCIPL and pRNFL, independent of a prior episode of optic neuritis [25, 26]. In addition, thinning is accelerated by ON episodes in MS patients, with most GCIP thinning occurring in the first months after ON onset, as described before [27]. Recent studies demonstrate conflicting data about the main focus of RNFL thinning. Depending on the cohort, temporal and nasal quadrants are most affected [28, 29]. Furthermore, progressive thinning of the retinal ganglion cell (RGC) and the inner plexiform layer has also been described for relapsing–remitting MS (RRMS) [29]. This more recent data is consistent with previous literature showing continuous RNFL thinning in CIS and MS patients with and without ON [14, 30, 31]. These findings are consistent with post-mortem analysis of MS patients, showing RNFL, GCL, and INL thinning [32]. The underlying pathophysiology of RNFL thinning is still in dispute. A loss of axons in the retina in patients with prior ON due to retrograde degeneration after an acute ON seems plausible. One possibility in patients without known prior ON is RNFL thinning after previous clinically silent ON episodes. Some papers discuss a direct autoimmune process involving retinal antigens. Another mechanism includes brain lesions in the anterior and posterior visual pathway leading to retrograde axonal degeneration or trans-synaptic degeneration of retinal cells and RNFL thinning. Correlation of lesion load in the optic radiation and RNFL thinning has been shown. Therefore, an effect of ongoing neurodegeneration and whole-brain atrophy on subsequent RNFL thinning seems possible [30, 31, 33–35].

GCIP and pRNFL thinning at disease onset, as well as during follow-up can be used to predict the conversion from radiologically isolated syndrome (RIS) and CIS to MS [36, 37]. In the first 5 years after MS onset, a thinning of pRNFL and GCIPL together with accelerated brain atrophy was found [38, 39]. Thinning is also increased by every relapse in RRMS patients. Disease progression without a relapse, typical for primary progressive (PPMS) or secondary progressive MS (SPMS), is also associated with increased GCIPL and pRNFL thickness loss [40]. This could, to a certain extent, explain why progressive MS patients show faster annual pRNFL and GCIPL thinning and lower values at baseline compared to RRMS [28, 41–43]. As mentioned before, GCIPL and pRNFL can be correlated with brain atrophy and spinal cord atrophy, cortical lesion volume, and leptomeningeal enhancement [44–48].

Consequently, an association between neurodegenerative markers and OCT parameters has been found. Serum neurofilament light chain (sNfL) and heavy chain (NfH) are inversely correlated with the outer plexiform layer and pRNFL thickness, respectively. The subgroup of SPMS patients showed higher NfH levels and lower pRNFL thickness compared to RRMS [49, 50]. In addition, reduced amyloid β levels in cerebral spinal fluid, a risk factor for worse clinical outcome in MS patients, is associated with reduced RNFL thickness [51].

Furthermore, OCT parameters have also been associated with disability in MS patients. OCT parameters correlating with disability include GCL thickness, pRNFL thickness, total macular volume, and annual atrophy rates of GCIPL and pRNFL [13, 14, 26, 52, 53]. GCIPL, pRNFL, and total macular volume are also associated with cognitive performance and executive function [54–56]. Consequently, GCIPL and pRNFL values at disease onset can be used to predict future disability and disease [43, 57, 58].

No correlation with OCT parameters was found for vitamin D levels, a common factor influencing MS [59]. In contrast, an influence of body mass index on GCIPL atrophy rates has been shown, specifically that obese patients exhibit faster atrophy compared to normal-weight patients [60]. A new OCT finding of unknown significance is the peripapillary hyperreflective ovoid mass-like structure (PHOMS). So far, it is mainly described as an accidental finding in different ophthalmologic diseases, and the underlying pathophysiology remains unclear. Furthermore, PHOMS is described in around 15–18% of MS patients, a higher prevalence than in healthy controls, and an association of PHOMS with the neurodegenerative activity in MS is speculated [61–63].

### OCT angiography in MS

OCT angiography (OCTA) is a newly emerging marker in MS research since a better understanding of the retinal blood vessels could lead to new pathophysiologic insights in ON. MS patients display a decreased vessel density in the radial peripapillary capillary plexus and the superficial capillary plexus (SCP). The effect on SCP is even more distinct in eyes with previous ON episodes [64–68]. The reduction in perfusion correlates with RNFL and GCL thinning after ON. While GCIPL thinning occurs early after ON, decreased vessel density of the SCP sets in 12 months later [69, 70].

A new approach measures the volumetric vessel density, calculated as the vessel density divided by the corresponding tissue. MS patients show an increased density with correlation to disability possible due to coexisting neurodegeneration and changes in the microvasculature [71]. There were no differences in the foveal avascular zone (FAZ) and macular and peripapillary vessel densities between MS patients and healthy controls [72].

Pathophysiological mechanisms leading to changes of the retinal vessels in MS patients remain unclear. A possible explanation could be the reduced lower metabolic activity due to thinning of the retinal layers and, therefore, downregulation of the vascular structures. Overall, OCT is a new method with only limited reports in the literature, but it can provide valuable data about the pathophysiology of ON and MS in the future [4].

### Effect of medication

Disease-modifying therapies for MS patients benefit disease course, relapse rate, and disability [73]. Since OCT parameters correlate with traditional outcome markers for MS in clinical studies such as disability or relapse rate, they are also of increasing interest in drug trials. A disease-modifying therapy with a known effect on the visual pathway is fingolimod. Fingolimod is known for causing macula edema as a side effect, requiring regular ophthalmological check-ups. The incidence for macular edema in real-life settings is about 1%, with good clinical outcomes after discontinuation of the medication [74]. Moreover, fingolimod mildly increases total macular volume in the general population [75, 76].

From the group of highly effective disease-modifying therapies, natalizumab showed a significantly reduced pRNFL thinning compared to first-line injectables [42]. While alemtuzumab exhibited stable RNFL and GCIPL parameters, proving a certain neuroprotective effect, rituximab showed increased GCIPL atrophy rates during the first 12 months of treatment but subsequently decreased atrophy rates similar to healthy controls in a study with a small case number [77]. Possible explanations of this effect include a late-onset neuroprotective effect of rituximab or pseudoatrophy due to decreased inflammation and therefore decreased edema after therapy initiation [78]. Mesenchymal stem cell transplantation showed, similar to alemtuzumab, a stabilization of RNFL thickness [79].

OCT lately became of increased relevance as an additional outcome marker for neuroprotective or remyelinating therapies. For example, epigallocatechin gallate, phenytoin, and clemastine showed no significant change of OCT parameters compared to controls [80–82]. Ibudilast, on the other hand, showed a decreased pRNFL and macula volume thinning and reduced brain atrophy [83]. Similarly, 4‑aminopyridine, commonly used to treat gait disturbances in MS patients, showed a reduced atrophy rate of the macular retinal fiber layer [84]. This means that both substances show promising neuroprotective effects, although further studies are necessary.

## Conclusion

OCT is currently used in MS patients with acute ON episodes to objectify symptoms and as an additional outcome parameter in clinical studies. Once more data become available and cut-off values are successfully established, it may become possible to confirm previous ON episodes using OCT. Progressing retinal layer thinning in MS has been well documented, but pathophysiological explanations and a more detailed look at different layers are still missing. Predicting the disease course for individual MS patients with OCT changes as sole parameters seems unlikely due to the complex nature of the disease. However, adding OCT parameters to a broader score, including clinical parameters, could be a promising concept in our opinion. More studies are necessary to gain deeper insights into the pathophysiological mechanism of ON and MS and OCT angiography. We expect to see an increase of OCT parameters in pharmaceutical studies on disease-modifying therapies and neuroprotective substances.
